# Ebola in the context of conflict affected states and health systems: case studies of Northern Uganda and Sierra Leone

**DOI:** 10.1186/s13031-015-0052-7

**Published:** 2015-08-08

**Authors:** Barbara McPake, Sophie Witter, Sarah Ssali, Haja Wurie, Justine Namakula, Freddie Ssengooba

**Affiliations:** Institute for International Health and Development, Queen Margaret University, Edinburgh, UK; Nossal Institute for Global Health, University of Carlton, Carlton, Australia; School of Women and Gender Studies, Makerere University, Kampala, Uganda; College of Medical and Health Sciences, University of Sierra Leone, Freetown, Sierra Leone; School of Public Health, Makerere University, Kampala, Uganda

**Keywords:** Ebola, Sierra Leone, Northern Uganda, Health System

## Abstract

Ebola seems to be a particular risk in conflict affected contexts. All three of the countries most affected by the 2014-15 outbreak have a complex conflict-affected recent history. Other major outbreaks in the recent past, in Northern Uganda and in the Democratic Republic of Congo are similarly afflicted although outbreaks have also occurred in stable settings. Although the 2014-15 outbreak in West Africa has received more attention than almost any other public health issue in recent months, very little of that attention has focused on the complex interaction between conflict and its aftermath and its implications for health systems, the emergence of the disease and the success or failure in controlling it.

The health systems of conflict-affected states are characterized by a series of weaknesses, some common to other low and even middle income countries, others specifically conflict-related. Added to this is the burden placed on health systems by the aggravated health problems associated with conflict. Other features of post conflict health systems are a consequence of the global institutional response.

Comparing the experience of Northern Uganda and Sierra Leone in the emergence and management of Ebola outbreaks in 2000-1 and in 2014-15 respectively highlights how the various elements of these conflict affected societies came together with international agencies responses to permit the outbreak of the disease and then to successfully contain it (in Northern Uganda) or to fail to do so before a catastrophic cost had been incurred (in Sierra Leone).

These case studies have implications for the types of investments in health systems that are needed to enable effective response to Ebola and other zoonotic diseases where they arise in conflict- affected settings.

## Introduction

Recent outbreaks of Ebola have disproportionately occurred in localities that have been badly affected by conflict in recent history. The inter-related civil wars affecting Liberia and Sierra Leone devastated the two countries throughout the 1990s, resulting in up to half a million deaths among less than 10 million people in the two countries combined, many more displaced and others co-opted into military service as children [1, 2]. The Liberian and Sierra Leonean conflicts spilled into Guinea resulting in an estimated 100,000 Guineans being displaced, 200,000 Sierra Leonean and Liberian refugees in Guinea in the early 2000s and some thousands of Guinean deaths.^1^ In 2004, there were still 59,000 refugees in the marginal environment of the forest region of Guinea, where the virus later emerged [3]. After the death of President Conté in 2008, Guinea fell into further governmental and civil disarray resulting in a series of coup d’états and periods of violence which have now subsided [3]. Other outbreaks, for example in Northern Uganda and in the Democratic Republic of Congo, where the disease is thought to have originated, have occurred in equally conflict-affected areas.

It is well understood that conflict and population displacement on a large scale poses significant risks for infectious disease, combined with livelihood disruption and food and water shortages [4]. Ford describes the associations between conflict and the re-emergence of African Trypanosomiasis in Sudan, the Democratic Republic of Congo (DRC), Angola and Uganda from the 1970s onwards [5], while Tong reviews the challenges of control during active conflict in DRC [6]. Bausch et al. [7] highlight the role played by political unrest in DRC in delaying a response to an outbreak of Marburg virus. Spiegel et al. [8] confirm a statistical association between the occurrence of natural disasters, complex emergencies and epidemics in the period 1995-2004.

War is one of a number of shocks that drives populations to marginal subsistence strategies with among many implications a likely increase in the recourse to bush meat and forest-based livelihoods. For example, crops may be deliberately or collaterally destroyed, people may temporarily or permanently lose access to land from which they normally derive subsistence or may otherwise become separated from their usual means of livelihood [9, 10]. These problems may last long into post conflict periods for example wherever population displacement persists. During conflict, active combatants may experience more short term exposure to forest conditions as they advance, retreat, conduct reconnaissance or engage in other war related purpose.

While it has been widely recognized that failure to manage the initial emergence of Ebola and prevent a full scale outbreak is premised on the ineffective operation of certain health system functions including early detection of cases, contact tracing, and timely response to outbreaks (for example [11]), if initial outbreaks are more likely in conflict-affected settings, strategies to both prevent and control outbreaks need to engage with the complexities of health and other systems in conflict-affected contexts. This paper focuses on published accounts of the two Ebola outbreaks of Northern Uganda (2000-2001) and West Africa (2014-15) and other work in progress in the two countries with which the authors are associated, which is building an understanding of the specificities of the health systems of conflict affected countries and working in both Northern Uganda and Sierra Leone (hence we focus on the Sierra Leone experience of the West African outbreak). It aims to identify the factors that contributed to the relatively successful control of the outbreak in Northern Uganda compared to Sierra Leone and to trace the extent to which both outbreak and control were affected by the histories of conflict in both settings.

## What do we mean by ‘conflict affected’?

The most used term among a set of terms that label states weak, dysfunctional or subject to the worst outcomes is the term ‘fragile’. This has been defined by DFID [12] as covering ‘those [states] where the government cannot or will not deliver core functions to the majority of its people, including the poor” ([8]:p7), and the OECD definition [13] is similar although more specific about the domains of core functions: ‘states are fragile when states lack political will and/or capacity to provide the basic functions needed for poverty reduction, development and to safeguard the security and human rights of their populations’. However these concepts of fragility are both imprecise and inclusive of highly heterogenous state characteristics [14]. To consider a subset of fragile states that are ‘conflict affected’ increases the specificity somewhat.

However, in the broadest sense, we are all ‘conflict affected’, either because the place we live has at some time been the location of conflict, because we are all interconnected inhabitants of a conflict-affected world, or because conflict is defined as encompassing the mildest of differences of opinion. It follows that the definition of conflict affectedness involves specifying time period, area whose inhabitants are affected, and the nature of behaviors that constitutes conflict. Moseley [15] defines *war* as ‘a state of organized open-ended collective conflict’ (p14), distinguishing it from a street brawl by the concept of organization; from a boxing match by the concept of open-endedness and from a personal feud by the concept of collectivity. While, as the author acknowledges, the definition remains incomplete and retains ambiguities, all these characteristics are consistent with our meaning of the term ‘conflict’ here. Brown et al. [16] recognize the complexity of the definition of ‘post-conflict’ situations and propose a typology based on milestones in the pathway from violent hostilities to economic recovery such as peace agreement, disarmament and demobilization, and the establishment of a functioning state, recognizing that movement occurs in both directions. Collier and Hoefller [17] have estimated that 40 % of countries relapse into conflict after a period of apparent recovery. We consider that ‘conflict-affectedness’ implies all intermediate points in the pathway and can (but need not) affect countries indefinitely.

## Health systems in stable and conflict-affected settings

Newbrander et al. [18] have proposed the following list of characteristics of post conflict health systems:

- Insufficient coordination, oversight and monitoring of health services
- Lack of equity in who receives the available health services
- Lack of mechanisms for developing, establishing and implementing national health policies
- Non-operational health information systems
- Inadequate management capacity
- Inability to provide health services to a large proportion of the population
- Ineffective or nonexistent referral systems
- Lack of infrastructure for delivering health services
- Nonexistent or inadequate capacity-building systems

This list is a fairly comprehensive account of characteristics that are common or typical of the health systems of low and even middle income countries as a whole, although the critical domain of human resource constraints is surprisingly absent. However, it might be argued that they are likely to be more acute in conflict-affected health systems to the extent that attempts to resolve such issues are likely to have stalled during active conflict periods in which other concerns may be considered more pressing. In addition, active conflicts such as wars may directly have affected elements such as infrastructure or management capacity, and governance mechanisms may have been diverted to provide oversight of the extremes of conflict-related activity.

Other characteristics have been proposed that suggest health systems in conflict-affected contexts can be differentiated qualitatively [19, 20]. Some of these are clear consequences of conflict and violence such as the desertion by health workers of conflict zones (where they are often at particular risk in excess of that of the general population), the damage to infrastructure and the loss of disease surveillance capabilities and records. The flight of health professionals from conflict affected states and areas does not only produce generalized shortages but skews those shortages to those areas that have been most insecure, often rural and remote ones and towards more clinical (emergency response) and away from public health functions. In some cases it creates a missing generation of health professionals, where there has been a prolonged period in which there has been no or little training and recruitment. Relatively inexperienced staff may find themselves in senior positions, for example managing national level programmes and negotiating with international agencies, which may undermine the ability of national institutions to lead the related agenda [21].

Post-conflict health systems can be inundated with aid in multiple forms as a country is considered to have a strong case for investment on the grounds of need (the country has for some time been unable to absorb aid effectively and the effects of war leave a legacy of humanitarian concerns) and on the grounds that investment may have the additional benefit of consolidating the peace process. This places requirements on often inexperienced officials to manage multiple aid-related actors including international NGOs, bilateral and multilateral agencies, all staffed by senior officials with extensive experience of other contexts, but quite often not experience of the country or region itself. Inevitably, these voices are more effective in directing policy agendas than local ones can be, even though they may be less able to understand the implications of policy advice in the specific context [21].

Furthermore, it has been argued (for example by Kruk et al. [4]) that there is an onus on health systems policies to communicate political and social values that promote reconciliation, inclusion and confidence in a new government. The extent to which the policies in practice succeed or fail in these respects assumes greater importance not only for health sector objectives but for the success of peace processes. The media may also play an important role here. It can be alarmist and can undermine government efforts as part of its political opposition, or it can support a public health strategy understood to be in the population’s best interest. Lowicki-Zucca et al. [22] provide three case studies of reporting in relation to HIV/AIDS in the context of conflict in Sudan, Uganda and Guinea and identify a series of stigmatizing, inaccurate and misleading statements, often repeated from previous news stories and argue that such reporting reinforces stigma and discrimination and ultimately increases the vulnerability of all populations irrespective of their relationship to the conflict.

Conflict affected health systems have to deal with increased levels of conditions associated with violence, infectious disease outbreaks that follow population disruption (of which Ebola may be an extreme example) and mental health problems that follow from the stress and dislocation associated with conflict [4].

## The Ebola outbreak in Uganda, 2000-1

Ebola emerged in the conflict-affected Gulu district of Uganda in October 2000, with two clusters first coming to the attention of authorities, among a group of funeral attendees, and among health staff at St Mary’s Lacor hospital, run by an Italian Roman Catholic mission in the district [23]. The index case was never identified, but it is thought that movement of people across the Ugandan, Sudanese and the Democratic Republic of Congo borders is likely to have been implicated [24], and it was speculated but never confirmed that the disease had been carried by Sudanese rebels operating in Gulu,^2^ or by the Ugandan military on return from Congo [25]. The potential for troop movements to be associated with disease outbreaks has also been demonstrated by the post-earthquake cholera outbreak in Haiti [26].

The disease infected 425 people, 224 of whom died, a case fatality rate of 53 %. 17 among the 224 dead were health workers. Centres for Disease control (CDC) [27], Lane and Nicoll [24], Okware et al. [28], and Lamunu et al. [23] provide descriptive accounts of the control response that by 16^th^ January 2001 had brought the outbreak officially under control, with the last confirmed case occurring on the 14^th^ January. The Ministry of Health was first alerted to the outbreak on 8^th^ October 2000 and a National Task Force (NTF) was established on 12^th^ October 2000 to coordinate the response; inter-ministerial and district task forces followed. Response strategies consisted of contact tracing, public education and community mobilization, isolation of cases, infection control through universal precaution and safe burial of the dead using trained burial teams. CDC established an on-site field laboratory for case confirmation. The NTF ensured that international support contributed to the agreed strategy and work plan: more than 25 international organizations contributed.

The cited accounts credit the success of the control efforts to a number of factors: prompt action and effective coordination at both national and district levels; conducive community protocols for dealing with infectious disease among the Acholi people [29]; effective public communication; use of trained burial teams to ensure safe burial in cases of suspicious deaths; effective surveillance mechanisms reaching into communities by the employment of community level scouts, trained for the purpose. The role of the media is highlighted by Okware et al. [28] as supportive of the control efforts, educating the public about control behaviours, reassuring and limiting panic, and explaining government actions such as quarantines. Media briefings from the National Task Force were organised twice per day. Despite newspaper stories reporting panic, stigmatizing reactions and a mix of likely effective and ineffective responses to the risk posed by the outbreak, Kinsman’s [25] account of the media’s coverage does not suggest that the media was itself a significant spur to these reactions. Moreover, it appears that the media was used strategically by the Ministry of Health as a means of advising the population on appropriate precautions and health providers on control measures.

The effective response may have been supported by the country’s successful management of its AIDS epidemic, widely attributed to its strategy of openness and leading to an understanding of the implications of being seen to hide information in public health emergencies. Uganda’s ‘open’ AIDS policy initiated in the 1980s encouraged officials at all levels to raise AIDS regularly at public meetings [25]. Uganda had also very recently controlled its largest recorded cholera outbreak that emerged at the end of 1997 [30] with likely significant opportunities for learning relative to the Ebola outbreak.

The control effort was not without problems. It was forced to operate in the context of continued conflict with army escorts employed to accompany surveillance teams to insecure areas [23] and the death of one scout during an attack on a medical team [25], although Okware et al. [28] report cooperation by rebel groups with the control effort in some instances. Supplies for barrier nursing were scarcely sufficient in the early phase of the outbreak and prior training had been insufficient [31] and 14 of 22 health workers were infected during the establishment of the first isolation ward [24] leading to the reinforcement of infection-control measures [27]. Health workers were not at first compensated for the additional risks or their families compensated for their deaths, until approximately two months into the outbreak [25].

Hewlett and Amola [29] describe the experience of the outbreak from the community’s perspective. The illness was first understood as any other, with recourse to a mix of biomedical and traditional treatments, the latter in particular responsible for significant use of family resources to secure traditional healers’ intervention. When this failed, the illness was reclassified in traditional terms, producing community responses including isolation of the sick, contact only by survivors of the illness or the elderly and constrained movement between villages, a set of responses that was highly consistent with those biomedically recommended. Burial practices were also changed in ways that may have reduced transmission, but only after the initial phase when normal burial practices were probably associated with a high level of transmission, particularly to women who are most exposed by traditional practices. Issues of trust between local communities and Euro-Americans may have affected the control effort as people sought to evade transport to hospital for fear of theft of their body parts if they died, initial post-mortems and sample collection having generated such rumours. The speed and lack of public visibility of burials conducted under control protocols contributed to this fear. Stigma also significantly affected survivors in ways likely to lead people to seek to conceal the early stages of illness [29, 24].

While some desertion from their posts by health workers is documented [31, 25] the more dominant behavior was to remain despite the risks and at times inadequate protection and this may reflect the reality that the health workforce already persisted in delivering services in the midst of conflict in which they were regularly at risk.

Life histories with health workers in the Acholi sub-region found that the Ebola outbreak of 2000-1 added to their ongoing challenges, such as experiencing injury and living under threat, with increased workloads and minimal professional support [32]. Those who stayed (largely mid-level cadres) were supported by encouragement from managers and elders, and appear to have been intrinsically motivated, taking pride in their work as health workers. Health workers coped with increased workload by taking on higher levels of responsibility than those they were qualified to do and by working in shifts. Allowances for outreach from NGOs to cope with epidemics such as Ebola also helped to keep them going in the absence of regular salaries, and other support from NGOs including unprecedented levels of training and engagement with international experts were also motivating [32, 33].

Despite the inevitable difficulties, Uganda appears to have managed well in the context of a conflict affected health system by providing good national leadership, likely benefiting from the national institutions in the central part of the country which had avoided recent conflict, enabling better coordination and oversight than might typically be expected. Uganda’s emergence from conflict at the national level was relatively immature, the outbreak occurring only 15 years after Yoweri Museveni had come to power and brought stability after a long period characterized by intermittent civil war and localized conflict. Nevertheless, its institutional development in that period has been considered rapid. Museveni’s first term until 2001 was strongly supported by foreign aid and the country experienced rapid economic growth [34, 35] While the North of the country generally failed to benefit from these trends [36] the availability of the national resources and institutions appear to have been a factor in the Ebola response.

As the conflict in the North was ongoing, the region was not at that stage significantly penetrated by a major international aid presence with concomitant concerns of co-ordination and sovereignty, thereby easing the coordination task. A major resource appears to have been provided by the St Mary’s Lacor hospital, a well-equipped, long standing and internationally supported and partially staffed facility. The standard of care and facilities at this hospital probably underpinned the comment of a WHO official that local facilities in Uganda were *‘outstanding compared to the classic Ebola situation’* [25].

Distrust of the national government, implicated in the regional conflict, appears to have constrained the control effort with respect to suspicions about the rationale for rapid and isolated burial and beliefs such as those that implicated Ugandan government forces in the origin of the outbreak. Overall, though, the national effort appears to have secured community acceptance in the main, and the success in controlling the outbreak may have contributed to confidence in the national government.

## The Ebola outbreak in Sierra Leone 2014-15

The 2014-15 Ebola outbreak in Sierra Leone originated in Guinea; the first case is thought to have been a child of 2 years in Guinea’s forest region in December 2013, most likely infected by a fruit bat. The disease had spread to health workers in the town of Guéckédou by January 2014 but the Ministry of Health was not notified until March, 2014 after which teams of health workers from both the Ministry of Health and the international non-governmental organization Médecins Sans Frontières (which was already present in the area through involvement in a malaria project) became involved. Blood samples were sent to Europe for analysis [37]. Until May 2014, cases in Liberia and Sierra Leone were very few but rose sharply at that point. By June 21^st^ 2015, 13,059 cases and 3, 928 deaths had resulted (case fatality rate 30 %)^3^.

Basic control measures, including early diagnosis with patient isolation, infection prevention control, contact tracing, barrier nursing, disinfection of contaminated objects and areas and safe burial appear to have been implemented slowly in all three countries due to shortages of staff, trained in infection prevention and control and lack of personal protective equipment, and limited laboratory, clinical management and surveillance capacities. Significant problems of lack of cooperation between population, national health system and international response teams also affected the success of control measures [38]. Deep rooted traditional beliefs played a part in the delays, as did lack of trust in the government and health system, which may be related to people’s experience of conflict,^4^ and of multiple decades of corrupt and partial treatment by government, especially with respect to ethnic and internal regional rivalries,^5^ both before and after the conflict [39]. The disease quickly moved across borders and to population centres where it was able to take root. High levels of population mobility – perhaps seven fold levels elsewhere in the world, have been implicated.^6^ The early media response in the region appears also to have been unhelpful, accusing the government of incompetence and corruption which, whether accurate or not, did not support public health measures. Government responses of attempts at media censorship compounded the difficulty^7^.^8^

Even after the situation was declared to be a Public Health Emergency of International Concern in August 2014, the response in Sierra Leone and its neighbours continued to be sluggish. In September, a total of 1,152 beds were estimated to be needed for Ebola cases in Sierra Leone, only 28 % were available and only an additional 25 % pledged to be set up by an identified partner. There were not even plans for 46 % of the estimated beds needed. Laboratory capacity was still in the process of being established; a mobile laboratory set up in Freetown had the capacity to undertake less than 100 tests per day; laboratories in the Northern and Southern regions were reported to be non-functional. Community level interventions were reported to be faring better with surveillance programmes reporting coverage of 90 % ‘*where contact tracing is being carried out’*; house-to-house campaigns reaching 75 % of all households; and burial teams operating in all affected districts.^9^

Despite recent improvements linked to the Free Health Care Initiative [40], the available health workforce is still inadequate and maldistributed and in most cases lacks the skills and training for the job at hand. Wurie and Witter [41] reported a chronically understaffed, demotivated, ill equipped, overworked health workforce in general, working in conditions described as poor, supported by inadequate logistics and resources and inadequate levels of health education and promotion. Also documented are non-payment or delayed payment of financial incentives implemented to motivate, attract and retain health workers in rural postings [41, 42]. This also emerged as an issue for concern as challenges were faced in the payment of risk allowances to frontline health care workers. In addition an enabling environment for practicing infection prevention and control was lacking. Collectively, these demotivating factors encouraged attrition from the health workforce and exacerbated the lack of trust between service users and health service providers. Service users were more accustomed to seeking health services from traditional healers instead of health facilities, particularly in the early phase of the outbreak.

Training pathways in Sierra Leone are clinically dominated and public health training is limited, implying that front line health workers had limited expertise in managing infection and that functions such as surveillance and contact tracing were largely unstaffed. Consequently, the majority of health specialists at the forefront, in the early phase of the outbreak were international experts flown in by international development partners, which delayed the response.

Sierra Leone was at a similar distance from national conflict in 2014 as Uganda in 2001, but it had recovered little from the paralysis of the economy and provision of public services and the destruction of infrastructure and governmental institutions wreaked by the conflict. Moreover, the conflict was linked to and compounded socio-economic and political divisions which predated it [43, 44]. Hanlon [45] describes the recreation of the political and economic conditions that fuelled the conflict in the post conflict settlement. The public health system in the aftermath of the conflict was practically collapsed. Only 16 % of the health centres were still functioning by 1996, mainly in Freetown [46]. While one of the tragedies of Ebola was that it came at a time when the health system was starting to recover - with reforms introduced since 2010 which reduced financial barriers for users while increasing support for and accountability of staff [40, 47], it was still badly affected by its pre- and post-conflict legacy. A ‘Service Availability and Readiness Assessment’ [48] found Sierra Leone lagging behind countries including Burkina Faso, Cambodia and Haiti in measures of health system capacity. The accumulated experience has led households to depend on informal and mixed care seeking, while from the health worker side, many staff were unofficial, working off payroll until recently and funded through informal payments from patients [40].

Another feature which appears to have hampered responses in Sierra Leone is the lack of decentralised authority within the health sector. Although decentralisation was initiated in 2004, core functions like hiring, deploying and managing staff remained highly centralised [41] impeding local responsiveness, a problem exacerbated by slow information and direction from the centre, as was the case in the Ebola outbreak. A recent study of district dynamics (pre-Ebola) highlighted the need to empower the DHMTs with the necessary tools to redress the power imbalances between them and the other actors, including international NGOs, at local level [49]. Some commentary suggests the possibility of the heavy NGO involvement in healthcare in Sierra Leone and Liberia being one of the reasons for the delay in the control of the epidemic because of the lack of investment in the meso-level of health sector administration [50].

A number of factors that are not clearly conflict related are also likely to have made control of the outbreak more difficult. The situation is thought to have been significantly complicated by the centre of the outbreak occurring amidst three international borders, giving rise to specific difficulties of co-ordination because the constellation of international agencies in each are markedly different, dominated by France in Guinea, the UK in Sierra Leone and the US in Liberia . International support to control the epidemic in the three countries whose systems were acknowledged to be weak failed to materialize. WHO ‘failed catastrophically’ in identifying, responding and alerting the international community to the outbreak ([51]: p1550). Peter Piot, one of the team that originally identified the Ebola virus in Zaïre (now DRC), is quoted as suggesting that it may have been scarred by its experience of the swine flu threat in 2009, when it was widely condemned for over-reaction, while others considered that the reaction was characterized by complacency . With earlier epidemics (the worst having been the Ugandan 2000-1 outbreak) quickly controlled, economic and political concerns may have been allowed to dominate public health ones.

## Conclusions

Conflict and its aftermath are among the factors that increase the opportunity for the Ebola virus to transmit from a forest animal to a human by disrupting livelihoods and living arrangements. Those whose normal subsistence is undermined, for example because their homestead is made insecure and those who are active conflict participants, appear to be at increased risk of being infected.

The disruption to public health systems that is a common effect of conflict undermines rapid case detection, contact tracing and quarantine arrangements. The uncertainties associated with the first Ugandan case in 2000 indicate the extent to which the virus had taken hold before it was recognized. Multiple theories about the index case implicating active combatants from either side and refugees, in turn, implicate the chaos associated with conflict at this stage.

The depleted state of health systems that are conflict-affected undermines control efforts. Damaged infrastructure, lost records, depleted health workforce and weak governance reflected in ineffective management, support systems, supplies systems and maldistribution of resources combine to frustrate effective public health intervention. The problems are worsened if international support is fragmented and poorly co-ordinated, or directed by people with little understanding of local context.

Table 1 compares a number of key features of the two cases studies suggesting that differences in local, national and international responses to the initial outbreaks are likely to have played a significant role in their different outcomes.

**Table 1:** A comparison of the features of the two case studies

	Uganda 2000-2001	Sierra Leone 2014-15
Cases	425	13059
Case fatality rate	53 %	30 %
Response features	National Task Force established within 4 days of MoH notification	Delay in notification of MoH
	CDC establishes local field laboratory	Slow implementation of control measures
	Effective co-ordination of international support	Sharp rise in cases 2 months after notification of MoH
		Basic control measures still absent 7 months into outbreak
Human resource factors	Significant difficulties but evidence of intrinsic motivation; supportive environment and positive role of international NGOs and experts.	Insufficient numbers, inadequate and inappropriate training and poor motivation all documented
Media	Effective use for public communication	Antagonistic relationship between government and press; accusations of government incompetence and attempts at censorship
	Limited scare mongering	
Community level	Some problems of stigma and distrust between community and health authorities but some community responses highly consistent with public health recommendations	Significant problems of lack of co-operation and trust, and conflict between public health measures and traditional practices
Institutional development	Rapid development in South of country in preceding 15 years provided basis for national institutional response	Limited economic and political recovery post conflict probably contributed to failures
	Importance of established faith based hospital	
International response	Fast, effective emergency response of agencies such as WHO and CDC	Delayed response may have been premised on complacency and political concerns.

The Uganda experience in 2000-1 shows that despite the unfavorable conditions of a conflict affected environment, effective containment and swift control can be achieved. Some features of the Ugandan case seem to contribute to an explanation of that success. One clear difference between it and the Sierra Leone case is that stable governance arrangements appear to have been re-established more effectively in the post conflict period of the South of the country allowing established national institutions of governance to oversee a competent response including effective co-ordination of international agencies. In contrast, aid co-ordination problems have been implicated as among those undermining control efforts in Sierra Leone and West Africa more generally.

At the centre of control efforts in Northern Uganda was St Mary Lacor hospital in Gulu, an NGO hospital that had established resilient systems capable of withstanding the operational difficulties of two decades of conflict around it. Its conflict related position, as a safe zone for people fleeing violence, contributed to the levels of trust that further strengthened its role during the outbreak. The faith based sector has also been identified as a key resource that has enabled effective response to Ebola outbreaks in DRC [52]. No equivalent hospital took on this role in Sierra Leone, with outbreak control reliant on government hospitals, affected by all the problems of weak governance and depleted resources.

Timing may also have mediated against a repeat of the Ugandan experience in West Africa. Both the explanations of complacency premised on previous successful control efforts, and of caution in relation to allegations of over-reaction following measures taken to manage the swine influenza outbreak of 2009 highlight path dependencies and factors that had affected global public health thinking between 2001 and 2014.

These case studies have implications for the types of investments in local, national and international health systems that are needed to enable effective response to Ebola and other zoonotic diseases where they arise in conflict- affected settings. At local level, building trust between communities and health providers relies on investment in a health workforce that is well supported financially, logistically and managerially and can establish strong relationships with service users. Understanding the importance of trust based relationships between community and health workers needs to inform reforms and projects with other intentions such as increasing accountability.

At national level, resources and technical support to those key functions that support local health workers require mechanisms to ensure local understandings of underlying issues are promoted, and effective co-ordination of international aid. The role of the media is important: public health authorities need to garner support for their actions from the media which can be an important component of the public education effort.

At international level, a re-prioritisation of infectious disease response readiness is required. The 2014-15 Ebola outbreak drew world attention to the strong interest that wealthy economies have in investing in the resolution of problems in weak states. The case studies help to identify the specific investments that are likely to be helpful but also highlight the complexity of the constellation of issues involved.

## End notes

1. http://www.globalsecurity.org/military/world/war/guinea.htm
2. Amarillo Globe News, October 20^th^, 2000: http://amarillo.com/stories/2000/10/20/usn_ebola.shtml
3. http://www.cdc.gov/vhf/ebola/outbreaks/2014-west-africa/case-counts.html
4. http://healthsystemsglobal.org/blog/14/Ebola-s-collision-with-the-Sierra-Leone-post-conflicthealth-system.html
5. http://www.economist.com/news/middle-east-and-africa/21610250-many-sierra-leoneans-refuse-take-advice-medical-experts-ebola-death
6. http://www.who.int/csr/disease/ebola/one-year-report/factors/en/
7. http://en.starafrica.com/news/sleone-legislators-summon-radio-manager-over-ebola-funds-reportage.html
8. http://www.reuters.com/article/2014/10/03/us-health-ebola-liberia-idUSKCN0HS15Q20141003
9. http://apps.who.int/iris/bitstream/10665/134771/1/roadmapsitrep_24Sept2014_eng.pdf?ua=1
